# Comparative analysis between open transvesical and laparoscopic adenomectomy in the treatment of benigne prostatic hyperplasia in a tertiary hospital in Curitiba-PR: a retrospective study

**DOI:** 10.1590/0100-6991e-20233450-en

**Published:** 2023-03-31

**Authors:** LUCIANO RICARDO SFREDO, ISABELLA CORREA DE OLIVEIRA, GUILHERME KYUZAEMON OSAKO NOVAKOSKI, INGRIDY DE SOUZA DIGNER, IVAM VARGAS MARTINS DA SILVA, DANIEL AUGUSTO MAUAD LACERDA, BRUNO CESAR MOLINA MALTAURO CAMPOS, LUIZ SÉRGIO SANTOS

**Affiliations:** 1 - Universidade Federal do Paraná, Departamento de Urologia - Curitiba - PR - Brasil; 2 - Universidade Federal do Paraná, Departamento de Cirurgia Geral - Curitiba - PR - Brasil; 3 - Universidade Federal do Paraná, Departamento de Medicina - Curitiba - PR - Brasil; 4 - Faculdades Pequeno Príncipe, Departamento de Medicina - Curitiba - PR - Brasil

**Keywords:** Urology, Prostatism, Prostatic Hyperplasia, Laparoscopy, Prostatectomy, Urologia, Prostatismo, Hiperplasia Prostática, Laparoscopia, Prostatectomia

## Abstract

**Objective:**

the aim of this study was to compare the results of open and videolaparoscopic transvesical prostatectomy techniques in the treatment of benign prostatic hyperplasia (BPH) in a tertiary hospital.

**Methods:**

we reviewed medical records of patients who underwent transvesical adenectomy due to BPH between March 2019 and March 2021 at the urology service of Hospital de Clínicas do Paraná (HCPR), 42 patients were included in the open transvesical prostatectomy group and 22 in the videolaparoscopic group. Then, a comparison was made between the techniques in terms of surgical time, bleeding, length of stay, need for intensive care, among others, in addition to postoperative outcome.

**Results:**

the mean surgical time was shorter in the open technique compared to the laparoscopic technique (141 min vs 274 min). The videolaparoscopic group had a shorter mean hospital stay (3.5 days vs 6.36 days). There was no statistical significance in the comparison regarding the need for an intensive care unit, as well as in the assessment of postoperative bleeding.

**Conclusion:**

comparatively, the techniques demonstrated a similar outcome, with a low rate of complications and satisfactory results for the treatment of BPH. The laparoscopic technique is a surgery with a shorter hospital stay, but at the expense of a longer surgical time.

## RESUMO

**Objetivo:**

o objetivo deste estudo foi comparar o resultado das técnicas de prostatectomia transvesical aberta e videolaparoscópica no tratamento de hiperplasia prostática benigna (HPB) em um hospital terciário.

**Métodos:**

foram revisados prontuários de pacientes submetidos a adenectomia transvesical devido a HPB entre março de 2019 a março de 2021 no serviço de urologia do Hospital de Clínicas do Paraná (HCPR), sendo incluídos 42 pacientes no grupo prostatectomia transvesical aberta e 22 no grupo videolaparoscópico. Em seguida foi feita a comparação entre as técnicas nos quesitos tempo cirúrgico, sangramento, tempo de internamento, necessidade de terapia intensiva, entre outras, além de desfecho pós-operatório.

**Resultados:**

o tempo cirúrgico médio foi menor na técnica aberta em comparação com a técnica videolaparoscópica (141 min vs 274 min). O grupo videolaparoscópico apresentou um tempo médio de internamento menor (3,5 dias vs 6,36 dias). Não houve significância estatística na comparação quanto a necessidade de unidade de terapia intensiva, assim como na avaliação do sangramento pós-operatório.

**Conclusão:**

comparativamente, as técnicas demonstraram um desfecho semelhante, com baixa taxa de complicações e resultados satisfatórios para o tratamento da HPB. Sendo a técnica videolaparoscópica uma cirurgia com menor tempo de internamento, porém às custas de um maior tempo cirúrgico. .

## INTRODUCTION

Benign prostatic hyperplasia (BPH) is the most common benign tumor in men^1^. This pathology manifests depending on different factors, such as the severity of the symptoms, prostate size, and general condition of the patient^2^. Therapeutic modalities vary from pharmacological therapies to surgical procedures, depending on the prostatic volume, such as transurethral resection of the prostate (TURP), open transvesical prostatectomy (OTP), or minimally invasive surgeries, such as enucleations or laparoscopic prostatectomy (LP)^3^.

According to American Urological Association (AUA) guidelines, surgery is indicated for patients with renal failure secondary to benign prostatic hyperplasia (BPH), patients with recurrent urinary tract infections (UTIs), bladder stones, or macroscopic hematuria due to BPH, and those who have lower urinary tract symptoms (LUTS) refractory to other therapies^4^.

The OTP technique was described and published for the first time in December 1947 by Millin^5^. It has three different approaches, retropubic, suprapubic, and perineal, the second being the one generally used in Brazil^4^.

While OTP has proven over the years to be an effective surgical approach, lowering the International Prostate Symptom Index (IPSS), there is ongoing research into less invasive treatment options, such as LP, due to significant complications of OTP, such as bleeding, need for blood transfusion, and revision surgery^6^.

The first LP was performed in 2006 and several subsequent series demonstrated functional results similar to those of the open technique^7^ ^,^ ^8^. Although these series have demonstrated the equivalence of performing a simple prostatectomy using a minimally invasive approach, this technique is still difficult to master and to teach^9^.

In this context, the objective of this study was to compare the OTP and LP techniques for the treatment of BPH in a medical residency service. As secondary objectives, we evaluated the epidemiological profile of patients undergoing OTP and LP and looked for factors related to perioperative surgical complications (decrease in hemoglobin/globular volume, need for ICU, transfusion, and surgical time) and postoperative outcome (length of stay, duration of catheterization, improvement of obstructive symptoms, and urinary incontinence).

## METHODS

In agreement with the ethics and research committee (CEP), under opinion 54125521.1.0000.0096, we carried out an observational and retrospective study that evaluated the 64 patients who underwent adenomectomy by the Urology department of the CHC-UFPR between March 2019 and March 2021, 42 in the OTP group and 22 in the LP group. The data source used was the review of medical records and the Hospital Information System. The variables analyzed were type of surgery, access, length of stay, drop in hemoglobin (HB), mean corpuscular volume (MCV), and need for blood transfusion. We also evaluated secondary variables related to demographic characteristics, such as age and comorbidities, to the disease, such as prostatic volume on digital rectal examination and ultrasound, and to treatment, such as need for transfusion or ICU in the postoperative period, evolution, and outcome. Statistical analysis was performed using the Student’s T, the Mann Withney, the Chi-square, and the Fischer tests.

## RESULTS

We included 64 patients in the study, 42 in the OTP group and 22 in the LP group. The age of the patients ranged from 52 to 85 years, the mean of the OTP group being 68 years and that of the LP group, 66. The mean prostatic volume in the open technique was 170g, with a minimum value of 85g and a maximum of 523g, and in the laparoscopic technique it was 127g, ranging from 85g to 205g, as shown in Table 1 (p=0.051). There was a tendency to use the open surgical technique in cases with a history of previous abdominal surgery, as well as in patients who had larger prostatic volumes as evidenced by ultrasonography.

**Table 1:** Distribution of demographic variables according to surgical technique (OTP VS LP) in absolute value and percentage.

		OTP		LP	
		Absolut value	Percentage (%)	Absolut value	Percentage (%)
AGE (years)	MEAN	68	-	66	-
BMI (kg/m2)	20-25	14	33%	6	27%
	25-30	17	40%	10	45%
	30-35	9	22%	4	18%
	35-40	1	3%	2	9%
	>40	1	3%	0	0%
		OTP		LP	
		Absolut value	Percentage (%)	Absolut value	Percentage (%)
Comorbities	SAH	28	66%	14	63%
	DM	9	21%	5	23%
	DLPM	2	5%	4	18%
	CKD	4	10%	0	0%
	OTHER	10	24%	2	10%
Previous abdominal surgery (p=0,103)	YES	16	38%	4	18%
	NO	26	62%	18	82%
Previous prostate surgery (p=0,337)	YES	5	12%	1	5%
	NO	37	88%	21	95%
Prostate volume on ultrassound (p=0,051)	MEAN (grams)	170	-	127	-

The most prevalent comorbidities were systemic arterial hypertension (43), type-II diabetes mellitus (14), and dyslipidemia (6). As for BMI, about 30% of the patients had adequate weight (20-24.9), 40% were overweight (25-29.9), and 20% of them were already considered obese grade I (30-34.9). Regarding previous surgical approaches, 68.8% of the patients had already undergone some type of abdominal surgery (p=0.103) and 9.4% had had some previous surgery on the prostate (p=0.307). There was no statistical significance in these analyses. All these variables were separated according to the surgical technique, in absolute value and in percentage, as shown in Table 1.

In the assessment of lower urinary tract symptoms (LUTS) before surgery, one patient had a low score (LP), seven were classified as moderate (4 OTP x 3 LP), 23 as severe (12 OTP X 11 LP) and 33 already had previous retention requiring the use of a catheter (26 OTP x 7 LP). Postoperatively, one patient was classified as severe (OTP), 6 moderate (4 OTP vs. 2 LP) and the others as mild (p=0.764).

In the evaluation of postoperative bleeding, we found no statistical significance when comparing the open and laparoscopic techniques. There was an average drop in HB, 1.97 vs. 2.26 (p=0.442), and MCV, 5.38 vs. 7.06 (p=0.139), respectively. Only one case required intraoperative transfusion (1 OTP x 0 LP, p=0.466).

The mean operative time was shorter in the open technique compared with the laparoscopic one (141 min vs. 274 min, respectively; p<0.001). However, the LP group had a shorter mean hospital stay (3.5 days vs. 6.36 days, p=0.034). There was no statistical significance for the need for an intensive care unit (2 OTP vs. 1 LP; p=0.969), nor for the average number of days with a catheter in the postoperative period (urethral or cystostomy) (13 days OTP vs. 11.3 LP; p=0.369).

There were six late complications (4 OTP vs. 2 LP; p=0.995), one case of hematuria and one of erectile dysfunction in the LP group, and one occurrence of urinary tract infection and three cases of surgical site infection in the OTP group. We observed urinary incontinence in 19% of the patients in whom the open technique was used and in 13% of the patients undergoing the laparoscopic technique, though without statistical significance (p=0.586). All complications presented were transitory, showing improvement of symptoms in the postoperative period.

## DISCUSSION

Comparative studies between transvesical and laparoscopic prostatectomy indicate that the two procedures are equivalent with regard to functional results and that laparoscopy brings benefits in the perioperative period. However, there are few relevant publications on the subject and with a significant number of patients^8^ ^,^ ^10^ ^,^ ^11^.

In this study, we evaluated 64 patients with BPH surgically treated at the institution, 42 (65.6%) undergoing OTP and 22 (34.4%) LP, with the focus on the perioperative and postoperative characteristics. As the results demonstrate, the larger prostates were approached by the OTP route. The choice for this surgical approach is probably due to the greater experience of surgeons at the service with the open technique, which is more consolidated and has a lower learning curve when compared with LP^11^. Thus, it was decided to allocate more complex and difficult cases to be performed via the OTP technique. However, this data was not statistically relevant (p=0.051).

**Figure 1:**
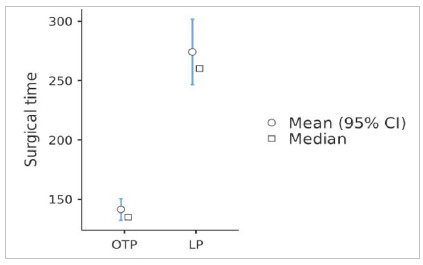
Surgical time by technique (p<0.001).

Regarding surgical time, our results are consistent the those found in the literature, since the average time to perform an OTP in this study was 141 min (median of 135 min), while for LP it was 274 min (median of 260 min), with p<0.001, as shown in Figure 1.

However, the results regarding blood loss disagreed with the findings reported in the literature, since there was a greater mean decrease in HB (1.97 OTP vs. 2.26 LP; p=0.442) and MCV (5.38 OTP vs. 7.06 LP; p=0.139) from preoperative measurement to the first postoperative day in the LP modality when compared with OTP.

**Figure 2:**
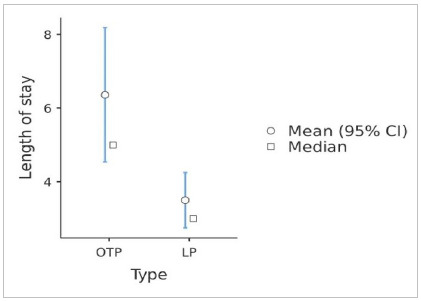
Length of stay by technique (p=0.034).

Another relevant data to be analyzed is the length of stay. In the present study, patients undergoing open prostatectomy had practically twice the average length of hospital stay when compared with those undergoing the laparoscopic technique (6.36 vs. 3.5 days, respectively), with a median of five days for OTP and three days for LP (p=0.034), according to Figure 2.

Comparative studies confirm that OTP and LP are comparable and equivalent techniques, with less bleeding, less need for transfusion, and shorter hospital stay in the second technique^8^ ^,^ ^10^ ^-^ ^12^. Porpiglia et al. presented the first comparative series and described minor bleeding in LP^8^. Baumert et al. compared 60 cases and also confirmed less bleeding, shorter irrigation time, and shorter hospitalization time in LP^11^.

Less bleeding reported in the studies is attributed to the CO_2_ pressure, which causes venous tamponade, and to the enlarged view of the endoscope, which allows careful enucleation and selective and continuous coagulation with the ultrasonic scalpel^13^ ^,^ ^14^. This is possibly due to the optimized vision of the LP, which guarantees a controlled enucleation, not being performed blindly as in the OTP. The length of hospital stay was shorter in the LP group, probably due to the classic advantages of laparoscopy: less pain, less need for analgesics, and less morbidity^15^.

Asimakopoulos et al. performed a critical analysis of the literature on LP, selecting 14 case series and three comparative studies comprising 626 patients, showing less bleeding, shorter stay, and shorter catheterization time at the expense of a prolonged surgical time^16^.

Garcia-Segui et al. observed a shorter catheter permanence time (5.5 days vs. 7.5 days, p=0.030), hospitalization (3.7 days vs. 6.6 days, p=0.006), and rate of transfusion (0% vs. 22.2%) in the laparoscopy group when compared with the open technique^12^.

Finally, another data evaluated was the improvement of lower urinary tract symptoms (LUTS) in the postoperative period. For this purpose, we used the International Prostate Symptom Score (IPSS), a questionnaire answered by the patient himself, consisting of seven items that assess storage and emptying symptoms, as well as a last item on quality of life^17^. Based on the responses, a score is calculated and the patient’s symptoms are classified as mild (0-7), moderate (8-19), and severe (20-35)^17^. In both treatment modalities, most patients had severe preoperative LUTS (29% OTP vs. 59% LP) or already had complications, such as previous episodes of acute urinary retention (62% OTP vs. 32% LP). In the postoperative evaluation, both groups had significant symptoms improvement, with low LUTS in 88% in the OTP and 90% in LP.

According to Garcia-Segui et al., the most common complications in the postoperative period of both surgical modalities are urinary tract infection/orchiepididymitis, hematuria, and surgical wound infection^13^. Other less important complications include hematoma, pulmonary edema, septic shock, acute myocardial infarction, urinary incontinence, acute urinary retention, and urinary fistula. In this work, the complications seen were one episode of hematuria and one of erectile dysfunction with the LP technique, and one episode of urinary tract infection and three episodes of wound infection with the OTP technique, all of which were transient and without statistical significance.

In addition to the biases inherent to retrospective studies, this study had other limitations, such as having taken place in a single center, a relatively small sample, and absence of sample size calculation. Thus, further studies on the subject are needed to better highlight the pros and cons of each type of approach.

## CONCLUSIONS

The analyzed variables showed results similar to what the literature found on the subject, with the exception of blood loss (without statistical significance). There was no statistical difference in prostatic volume, previous surgeries, need for blood transfusion, postoperative need of ICU, complication rate, and urinary incontinence.

Comparatively, both techniques showed a similar outcome, with a low rate of complications and effective results for the treatment of BPH. Notably, however, LP demonstrated a shorter hospital stay, though at the expense of a longer surgical time.
